# An Elementary Formula for the Initial Relaxation Modulus from the Creep Compliance for Asphalt Mixtures

**DOI:** 10.3390/ma16186097

**Published:** 2023-09-06

**Authors:** Songqiang Chen, Bin Chen, Xi Wu, Jian Zhou

**Affiliations:** 1 Zhejiang Engineering Research Center of Intelligent Urban Infrastructure, Hangzhou City University, Hangzhou 310015, China; chensongqiang@zucc.edu.cn (S.C.); chenbin@zucc.edu.cn (B.C.); 2 Balance Building Research Center, Zhejiang University, Hangzhou 310058, China; 3 Zhejiang Yangtze River Delta Infrastructure Research Institute, Hangzhou 310015, China; 4 Key Laboratory of Safe Construction and Intelligent Maintenance for Urban Shield Tunnels of Zhejiang Province, Hangzhou City University, Hangzhou 310015, China; zhoujian@zucc.edu.cn

**Keywords:** asphalt mixtures, viscoelasticity, creep compliance, initial relaxation modulus, interconversion

## Abstract

The conversion between the relaxation modulus and creep compliance is a traditional research topic in viscoelastic materials. Generally, different methods have been used to solve the numerical solution based on convolution theory. However, the initial relaxation modulus (relaxation modulus at *t* = 0) has been difficult to obtain. This paper aimed to propose a fast calculation method to derive the initial relaxation modulus from the creep compliance. First, three groups of uniaxial static creep tests of asphalt mixtures were conducted to determine the creep compliance of the experimental data. Then, the calculation of the initial relaxation modulus from the creep compliance by three inversion methods (midpoint method, approximate method, and Laplace numerical inversion method) was evaluated. The results indicate that approximate method and Laplace numerical inversion method cannot calculate the initial relaxation modulus value, and the calculation results of the midpoint method can only approach the exact value infinitely, for which calculating the relaxation modulus at 0.0005 s requires 2000 s. The results can only approach the exact value infinitely and take a lot of computing time. Finally, a fast calculation method for the initial relaxation modulus is proposed and verified by Laplace initial value theorem, and this method can directly derive a simple expression for calculating the initial relaxation modulus without requiring computational time. The proposed calculation methods of the initial relaxation modulus for various viscoelastic models were then put forward. The research results provide an effective tool for obtaining the initial relaxation modulus accurately.

## 1. Introduction

Viscoelastic materials are widely used in aerospace, automotive industry, civil engineering structures, and some new applications (biomechanics) due to their excellent properties. Viscoelastic material parameters such as creep compliance, relaxation modulus, and dynamic modulus are important evaluation indices of asphalt mixtures properties [1,2,3,4]. R. Christensen [5] and Findley [6] systematically analyzed the mechanical properties, thermodynamic properties, and nonlinear viscoelastic properties of viscoelastic materials in their work. Due to the lack of testing instruments, some viscoelastic parameters could not be measured by the testing instruments directly and needed to be calculated by the interconversion relationship of viscoelastic material parameters. Thus, parameter conversion of viscoelastic materials has become an important research topic, especially the relaxation modulus from creep compliance [7,8].

Hopkins and Hamming [9] proposed a numerical calculation method for the conversion of the relaxation modulus and creep compliance based on the convolution formula, where the finite integral is discretized into subinterval integrals and the trapezoidal integral formula is used to obtain the relaxation modulus of each subinterval. Park and Schapery [10,11] presented numerical and approximate solutions for viscoelastic parameter conversion by the Prony series and proved that these methods were suitable for viscoelastic solids and fluids. Liu [12] adopted the nonnegative least square method to obtain the relaxation modulus time spectrum directly from creep test data and then constructed the relaxation modulus calculation model. Sungho Mun [13] employed the approximate calculation method and a Wiechert model of the relaxation function to convert the viscoelastic parameters in the time domain and frequency domain based on the stress–strain relationship in linear viscoelastic theory. M Saleh [14] used the Tikhonov regularization method [15] (Hansen 1998) and the L-curve to determine the relaxation modulus from creep compliance based on the convolution integral. The Tikhonov regularization method is one of the most common and widely used regularization methods and has been successfully applied in the conversion of viscoelastic material parameters [16,17,18]. Hajikarimi P [19] obtained the relaxation modulus in time-domain solution by Laplace transform by expressing the creep compliance as a power-law function. Katsourinis S [20] employed fractional viscoelastic models to study the static and dynamic viscoelastic parameters of different polymers. Zhang [21] uses numerical calculations to convert time–domain and frequency–domain parameters into each other. Zeng and Zhang [22,23,24] analyzed the configuration, multi data, and window methods to establish a discrete relaxation/retention spectrum conversion relationship and proposed a hybrid solution method to achieve viscoelastic parameter conversion. Xi [25] proposed a model based on the storage modulus and loss modulus models to derive the same model parameters and ultimately determine the continuous relaxation spectrum for viscoelastic parameter conversion. These conversion methods all use the mathematical calculation method to convert viscoelastic parameters based on the relationship between the material parameters. However, these methods require a complex mathematical derivation process and strong programming ability.

In this paper, an elementary formula for the initial relaxation modulus from the creep compliance is proposed. Firstly, the viscoelastic mechanics and the relationship between viscoelastic parameters were reviewed. Then, the creep compliance of the asphalt mixture was obtained by a static loading test. Afterwards, the application of three conversion methods for creep compliance and relaxation modulus in the conversion of asphalt mixture parameters was discussed, and the difficulties of three methods in solving the initial value of relaxation modulus were analyzed. Finally, an elementary formula for the initial relaxation modulus from the creep compliance was proposed, and the calculation methods of the initial relaxation modulus for various viscoelastic models were proposed.

## 2. Linear Viscoelastic Theory and Parameter Conversion Method

### 2.1. Linear Viscoelastic Theory

**Figure 1:**
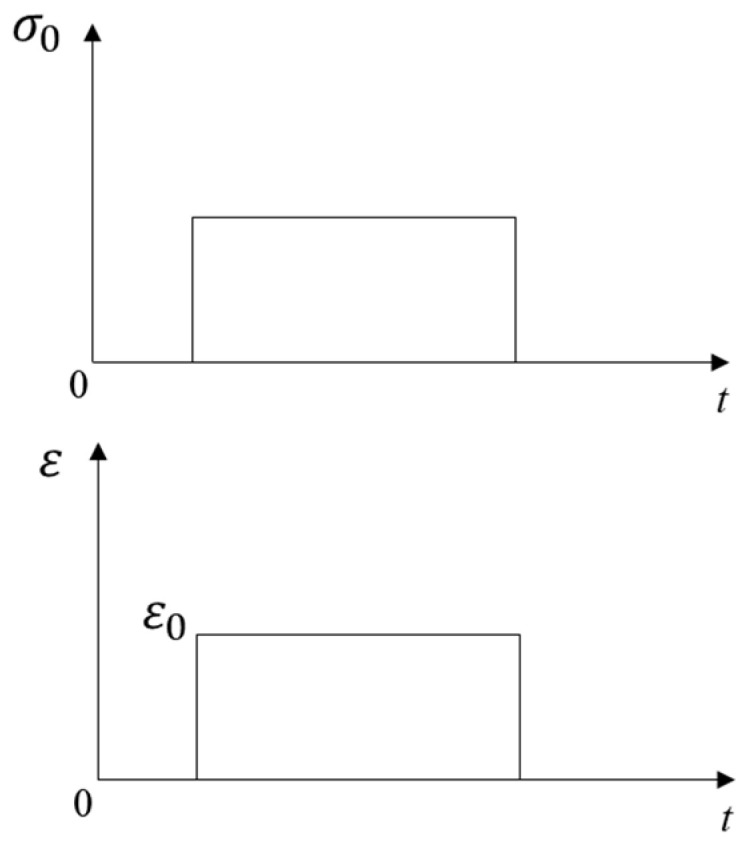
Stress–strain relationship of elastic materials.

**Figure 2:**
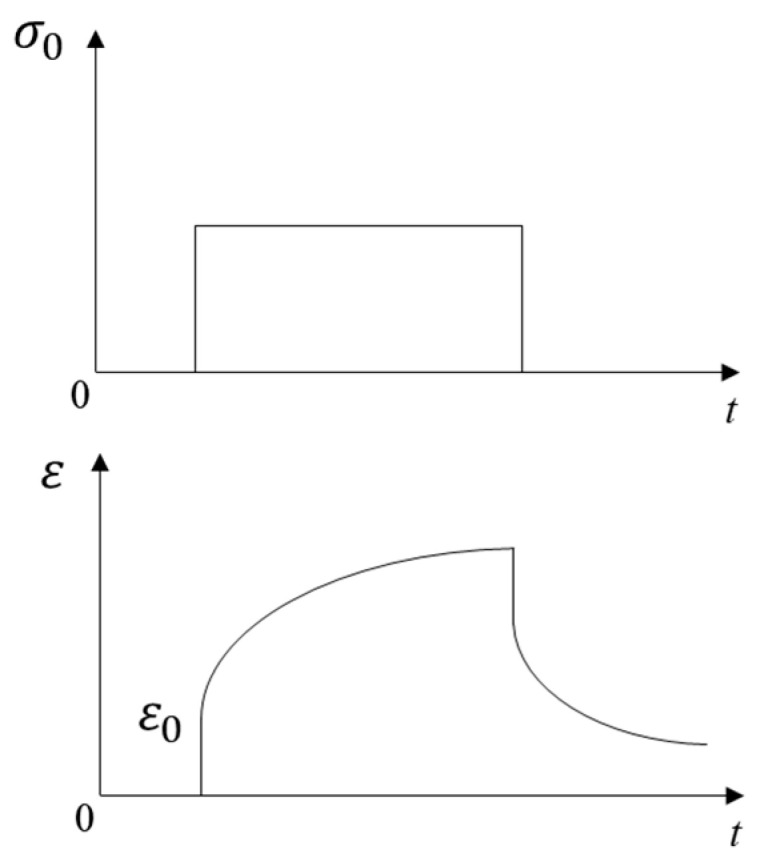
Stress–strain relationship of viscoelastic materials.

**Figure 3:**
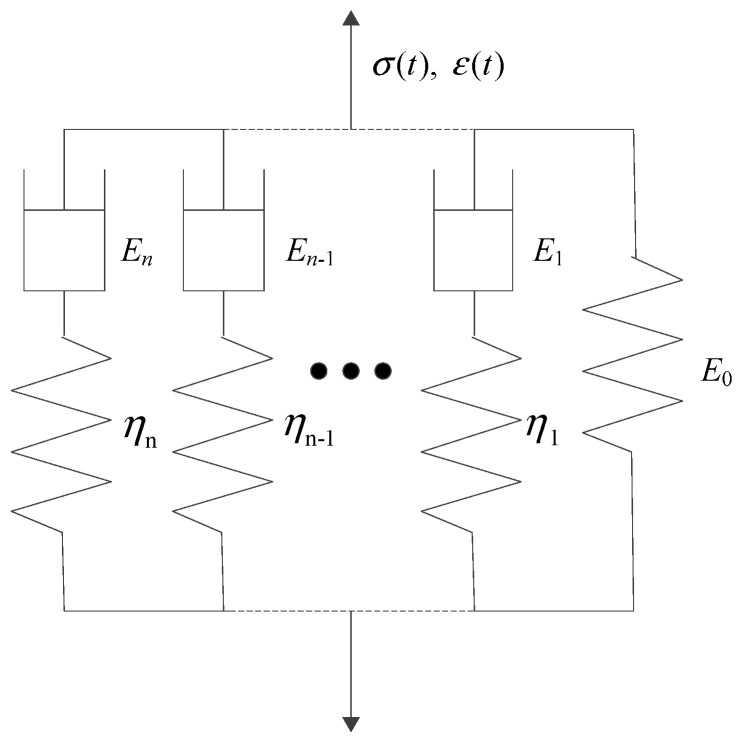
Generalized Maxwell model.

**Figure 4:**
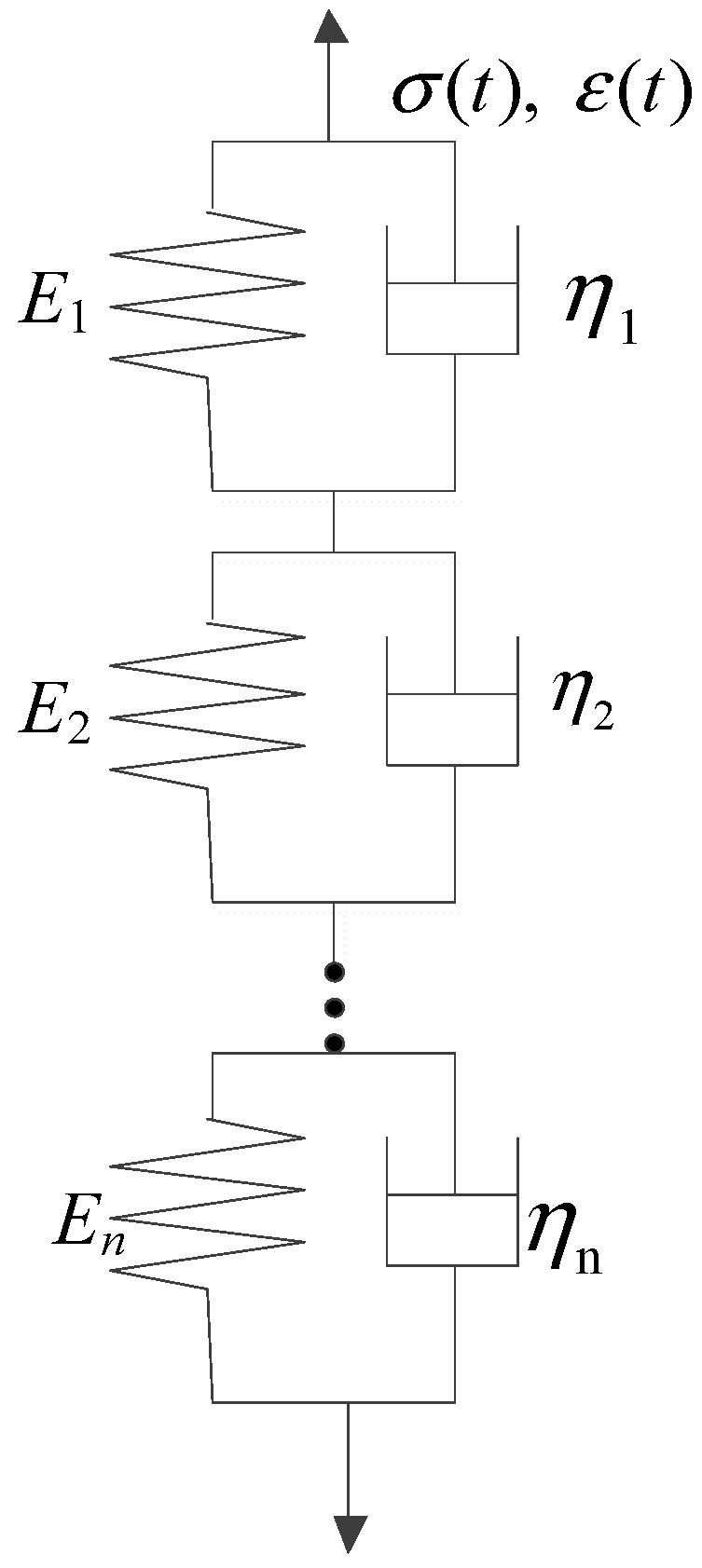
Generalized Kelvin model.

The stress–strain relationships of elastic and viscoelastic materials with time are shown in Figure 1 and Figure 2, respectively. The stress–strain relationship of elastic materials is linear, and material parameters are easily converted to each other. The stress–strain relationship of viscoelastic materials is complex, and the correlations between material parameters are also relatively complicated. The stress–strain relationship of linear viscoelastic materials can be divided into two categories: differential and integral. The classical viscoelastic constitutive model is composed of a spring and a viscous pot in series or in parallel, from which different constitutive models were formed, such as Maxwell, Kelvin, and Burgers models. The generalized Kelvin model and generalized Maxwell model were composed of *n* Kelvin models or Maxwell models, respectively, as shown in Figure 3 and Figure 4. The generalized Kelvin model is used to analyze the derivation process of viscoelastic parameters.

The strain of the *i*th element of the Kelvin chain is assumed to be $\varepsilon_{i}$, where the elastic modulus of the spring and viscous coefficient of the damper are $E_{i}$ and $\eta_{i}$, respectively, and the strain is expressed as follows:

(1) $$\varepsilon_{i}=\frac{\sigma }{E_{i}+\eta_{i}D}$$

Therefore, the total strain of the generalized Kelvin model consists of the strains of *n* Kelvin models. The total strain can be calculated as follows:

(2) $$\varepsilon =\sum_{i=1}^{n}\varepsilon_{i}=\sum_{i=1}^{n}\frac{\sigma }{E_{i}+\eta_{i}D}$$

The constitutive equation of the generalized Kelvin model can be obtained by sorting out the expansion as follows:

(3) $$p_{0}\sigma +p_{1}\overset{•}{\sigma }+p_{2}\overset{••}{\sigma }+p_{3}\overset{•••}{\sigma }+\cdots =q_{0}\varepsilon +q_{1}\overset{•}{\varepsilon }+q_{2}\overset{••}{\varepsilon }+q_{3}\overset{•••}{\varepsilon }+\cdots$$

Then, Equation (3) can be written as follows:

(4) $$\sum_{k=0}^{m}p_{k}\frac{d^{k}\sigma }{dt^{k}}=\sum_{k=0}^{m}q_{k}\frac{d^{k}\varepsilon }{dt^{k}}$$

Equation (4) can be simplified as follows:

(5) Pσ=Qε

where $P=\sum_{k=0}^{m}p_{k}\frac{d^{k}\sigma }{dt^{k}}$, $Q=\sum_{k=0}^{m}q_{k}\frac{d^{k}\varepsilon }{dt^{k}}$.

As is well known, the Laplace transform is an important mathematical tool to solve viscoelastic problems. The Laplace transform made the elastic problem and viscoelastic problem consistent. Thus, the viscoelastic problem could be solved by the elastic method [26,27,28], which is named the principle of elastic–viscoelastic correspondence. The Laplace transform is applied to Equation (5), and the expression is in the following form:

(6) $$\bar{P}(s)\bar{\sigma }(s)=\bar{Q}(s)\bar{\varepsilon }(s)$$

where $\bar{P}(s)=\sum_{k=0}^{m}p_{k}s^{k}$, $\bar{Q}(s)=\sum_{k=0}^{m}q_{k}s^{k}$, and *s* is the variable of Laplace transform.

For the static creep test, the load is fixed, which could be expressed as $\sigma =\sigma_{0}H(t)$, as shown in Equation (7). The Laplace transform of $\sigma =\sigma_{0}H(t)$ is $\bar{\sigma }(s)=\frac{\sigma_{0}}{s}$. Then, $\bar{\sigma }(s)=\frac{\sigma_{0}}{s}$ is substituted into Equations (6) and (8) is obtained.

(7) $$H(t)=\left\{ \begin{array}{l} 1\;\;t\geq 0\\ 0\;\;t<0 \end{array}\right.$$

where H(t) is the unit impulse response.

(8) $$\bar{P}(s)\frac{\sigma_{0}}{s}=\bar{Q}(s)\bar{\varepsilon }(s)$$

Therefore, the creep compliance *J*(*s*) in the Laplace domain can be expressed in the following form:

(9) $$J(s)=\frac{\sigma_{0}}{\varepsilon (s)}=\frac{\bar{P}(s)}{s\bar{Q}(s)}$$

To obtain the solution of the relaxation modulus, the Laplace integral transform of the strain function is substituted into Equation (6), and the solution of the relaxation modulus in the Laplace domain *E*(*s*) is obtained as follows.

(10) $$E(s)=\frac{\varepsilon_{0}}{\sigma (s)}=\frac{\bar{Q}(s)}{s\bar{P}(s)}$$

The following equation can be obtained from the product of Equations (9) and (10):

(11) $$E(s)J(s)=\frac{1}{s^{2}}$$

The convolution formula of creep compliance and relaxation modulus can be obtained by applying the inversion of the Laplace transform to Equation (11) as follows:

(12) $$\int_{0}^{t}E(t-\tau )J(\tau )d\tau =t$$

where τ is the time variable.

At present, most studies on the interconversion of creep compliance and relaxation modulus are based on Equation (12), which is considered an ill-posed problem [29]. This means that the solution of this kind of equation is not unique. In some studies, the integral is discretized into the sum of finite section integrals. By assuming the relaxation modulus of each section as a fixed value, the numerical integration method is used to solve the relaxation modulus value of each section [19,30]. In other methods, the parameters of the relaxation modulus model were obtained by matrix operation on the basis of the convolution relation by assuming the mathematical model of creep and relaxation modulus [31,32]. In addition, Chen [33] derived the relaxation modulus calculation formula in the Laplace domain based on Equation (11), and then the time–domain solution of the relaxation modulus is calculated by the Laplace numerical inversion method. R J Loy [34] obtained the relaxation modulus mathematical model parameters through the differential expression and matrix operation of creep compliance in the Laplace expression domain. All these methods need to use numerical calculation methods.

### 2.2. Parameter Conversion Method

The conversion calculation methods of creep compliance and relaxation modulus can be divided into three categories: midpoint method, approximate interconversion method, and Laplace transform method.

(1) Midpoint method

In this method, the integral of Equation (12) is discretized into *n* equal integral intervals as follows:

(13) $$\int_{0}^{t}E(\tau )J(t-\tau )d\tau =\sum_{i=1}^{n}\int_{t_{i-1}}^{t_{i}}E(\tau )J(t-\tau )d\tau =t$$

Since Equation (13) is an ill-posed equation, the exact solution of relaxation modulus E(τ) cannot be obtained. Therefore, the relaxation modulus in each integral interval is assumed to be a constant value and replaced by the midpoint value $E(\frac{t_{i}+t_{i+1}}{2})$. Equation (13) can be written in the following form:

(14) $$\sum_{i=1}^{n}\int_{t_{i-1}}^{t_{i}}E(\tau )J(t-\tau )d\tau =\sum_{i=1}^{n}E\left(\frac{t_{i-1}+t_{i}}{2}\right)\int_{t_{i-1}}^{t_{i}}J(t-\tau )d\tau =t$$

In this way, the numerical integration method can be used to obtain the integral value of each interval,$\int_{t_{i-1}}^{t_{i}}J(t-\tau )d\tau$, and then the relaxation modulus value of the midpoint of each integration interval could be calculated based on Equation (14).

(2) Approximate interconversion method

The approximate interconversion method is generally based on Equation (11) or Equation (12) and inverses the relaxation modulus by using the approximate value of the relation. Ferry [35] employed the mathematical model of Equation (15) to represent the creep compliance. Then, the Laplace transform is applied to Equation (15) and obtains Equation (16). Equation (16) is substituted into Equation (11) and the relaxation modulus in the Laplace transform domain is derived as shown in Equation (17). Then, the Laplace inverse transform is applied to Equation (17) and the relaxation modulus in the time domain is derived as shown in Equation (18). Finally, the approximate calculating formula is shown in Equation (19) is obtained with the approximate formula of Γ(n)Γ(1−n)=π/sinnπ and Γ(n+1)=nΓ(n). Denby [36] proposed another calculation method based on an approximation of $\Gamma (n+1)\Gamma (1-n)=1/(1+(n^{2}\pi^{2}/6))$, and the relaxation modulus in the time domain could be derived as shown in Equation (20):

(15) $$J(t)=J_{1}t^{n}$$

(16) $$J(s)=J_{1}\frac{\Gamma (n+1)}{s^{n+1}}$$

where Γ() is the gamma function and *s* is the variable of the Laplace transform.

(17) $$E(s)=\frac{1}{J_{1}\Gamma (n+1)s^{1-n}}$$

(18) $$E(t)=\frac{t^{-n}}{J_{1}\Gamma (n+1)\Gamma (1-n)}$$

(19) $$E(t)=\frac{t^{-n}}{J_{1}n\Gamma (n)\Gamma (1-n)}\approx \frac{\sin(n\pi )t^{-n}}{J_{1}n\pi }$$

(20) $$E(t)\approx \frac{t^{-n}}{J_{1}(1+(n^{2}\pi^{2}/6))}$$

(3) Laplace transform method

The Laplace method avoids the tedious numerical integration process. The Laplace domain solution of the relaxation modulus is obtained through the interconversion between the relaxation modulus and creep compliance in the Laplace domain, and then the time domain solution of the relaxation modulus could be solved by the Laplace inverse numerical transform.

Chen [22] first adopted the Prony series to fit creep test data. Then, the Laplace transform is applied to the Prony series, and Equation (21) is obtained in the following form:

(21) $$J(s)=\frac{J_{0}}{s}+\sum_{i=1}^{7}J_{i}(\frac{1}{s}-\frac{\tau_{i}}{\tau_{i}s+1})$$

where *J*(*s*) is the Laplace transform of *J*(*t*) and *s* is the variable of the Laplace transform.

The formula for the relaxation modulus in the Laplace domain is obtained by substituting Equation (21) into Equation (11) as follows:

(22) $$E(s)=\frac{1}{J_{0}s+\sum_{i=1}^{7}J_{i}(s-\frac{\tau_{i}s^{2}}{\tau_{i}s+1})}$$

The key to the Laplace inversion method is to select an inversion algorithm with high accuracy and efficiency. The fixed Talbot method (FT method) [37], a numerical Laplace transform method based on the deformation of the contour of the Bromwich inversion integral, is selected to apply to Equation (22) to calculate the relaxation modulus in the time domain. The result [22] shows that the error is less than 10^−4^, and the computational speed is increased by 25 times compared with the results of convolution.

(23) f(t)=rM{12F(r)ert+∑k=1M−1Re[et⋅s(θk)F(s(θk))(1+iσ(θk))]},

where f(t) is the time–domain solution via the inverse Laplace transform and F(x) is a function expression in the Laplace domain, corresponding to F(s) of Equation (22), Re[*x*] is the real part of the complex data, *i* is the representation of the imaginary part of a complex number, *t* is time, s(θ)=rθ(cotθ+i), σ(θ)=θ+(θcotθ−1)cotθ, r=2M/5t, and $\theta_{k}=k\pi /M$.

## 3. Material and Testing

Three types of asphalt mixtures, fabricated by using three kinds of asphalt and gradation, were utilized in this study for laboratory experiments. The asphalt properties and mixture gradations, SMA-13, AC-20, and ATB-25, are shown in Table 1 and Table 2. SBS rubber compound-modified asphalt, SBS-modified asphalt, and 90# asphalt were used to fabricate SMA-13, AC-20, and ATB-25, respectively. The optimum asphalt contents were 6.0%, 4.5%, and 3.8%, determined by the Marshall test, and the result was shown in Table 3. The asphalt mixture specimens with a diameter of 150 mm and a height of 170 mm were compacted by a superpave gyratory compacter (SGC), and standard specimens with a diameter of 100 mm and a height of 150 mm were obtained by the coring machine.

**Figure 5:**
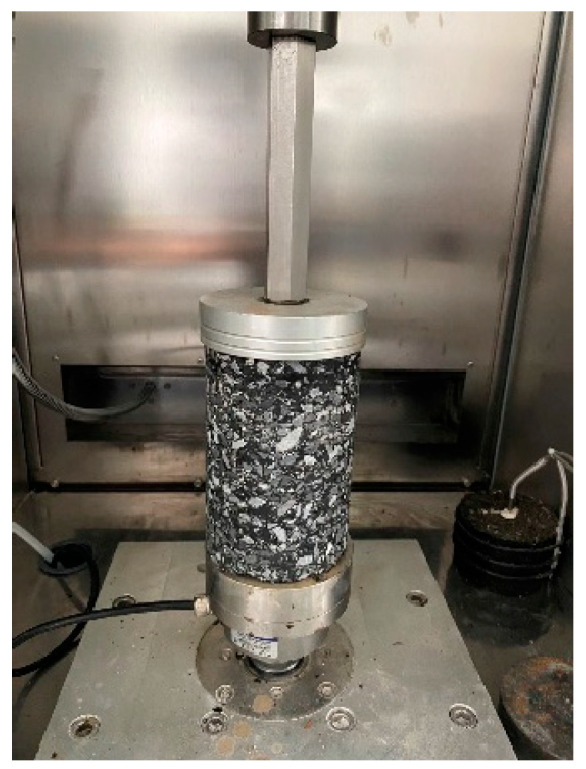
Creep tests.

The uniaxial compressive creep tests, as shown in Figure 5, were conducted on a united testing machine (UTM) at ambient temperatures of 20 °C and 35 °C and loads of 0.1 MPa and 0.3 MPa. To reduce the influence of the environment and load on the test results, the specimens were stored at a constant temperature for 4 h, and the specimens were preloaded at 50 kPa for 30 s before the test. The loading time was 240 s. The displacement sensor was used to collect the creep displacement of the specimen during the loading process, and the creep compliance was determined as follows:

(24) $$J(t)=\frac{\Delta l}{h\sigma_{0}}$$

where *J*(*t*) is creep compliance (MPa^−1^), Δl is the deformation of the specimen (mm), *h* is the height of the specimen (mm), and $\sigma_{0}$ is the applied load (MPa).

**Figure 6:**
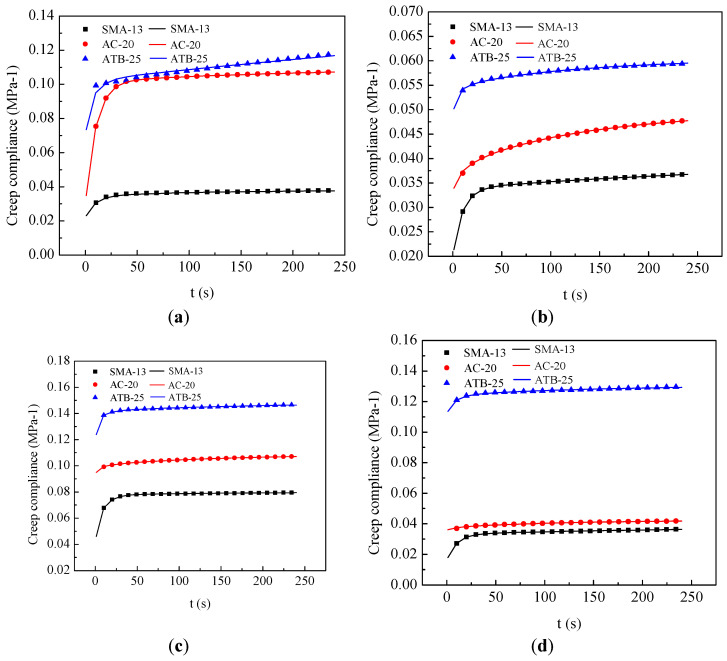
Creep compliance of asphalt mixtures under different temperatures and loads. (**a**) 0.1 MPa, 20 °C. (**b**) 0.3 MPa, 20 °C. (**c**) 0.1 MPa, 35 °C. (**d**) 0.3 MPa, 35 °C.

Figure 6 presents the creep compliance of three types of asphalt mixtures at 20 °C and 35 °C and 0.1 MPa and 0.3 MPa. The creep compliance obviously increases and the growth rate obviously decreases with increasing loading time. It can be seen from the figure that the creep compliance of SMA-13 is the smallest, followed by AC-20, and ATB-25 is the largest, indicating that SMA has the strongest deformation resistance, followed by AC-20, and ATB-25 is the worst. In addition, it can be seen that the creep compliance of asphalt mixture increases, and its deformation resistance decreases as the temperature increases.

**Figure 7:**
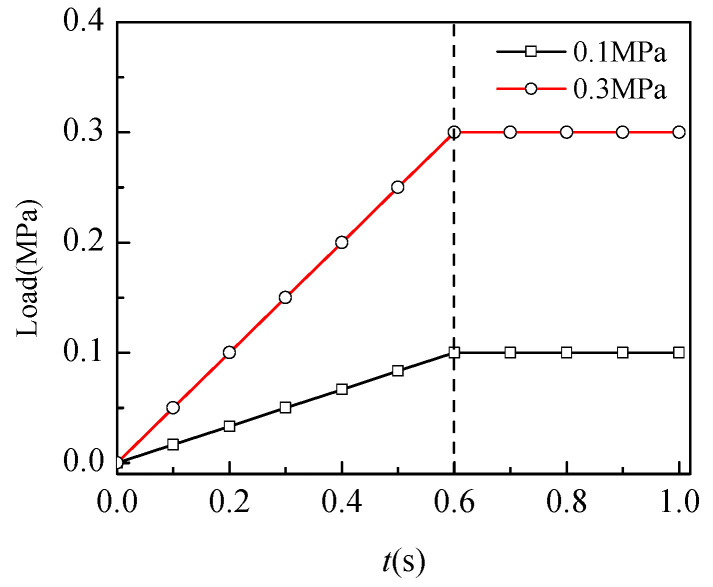
Initial loading stage.

The Prony series and power law were utilized to model the creep compliance experimental data. The Prony series is the simplified generalized Kelvin model, as shown in Equation (14). The retardation times employed a fixed constant, as shown in Table 4. The power law, as shown in Equation (26), is an effective mathematical model of viscoelastic parameters, and the form is shown in Equation (26). Parameters *J*_0_ and *a* refer to the response of viscoelastic materials at the moment of loading. Generally, it took time for the test instrument to load to the set load, and this time point was used as the initial moment to determine the value of *J*_0_ and *a* based on the stress–strain relationship at that time point. It can be seen from Figure 7 that the initial moment time is 0.6 s, and the parameters *J*_0_ and *a* were determined by the mechanical response at *t* = 0.6 s. The curve fitting of the MATLAB (Matrix Laboratory 2018b) tool was utilized to obtain the coefficients of the Prony series and power law, as shown in Table 5, Table 6, Table 7 and Table 8.

(25) $$J(t)=J_{0}+\sum_{i=1}^{7}J_{i}(1-e^{-t/\tau_{i}})$$

where *J*(*t*) is the creep compliance, *J_i_* are coefficients of the Prony series, $\tau_{i}$ are the retardation times, and t is the time.

(26) $$J(t)=a+bt^{m}$$

where *a*, *b*, and *m* are the parameters of the power law.

## 4. The Existing Interconversion Methods and Discussion

### Evaluation of Existing Methods for Solving the Initial Relaxation Modulus

The midpoint method, approximate method, and Laplace transform method are three categories for the interconversion of the relaxation modulus and creep compliance. However, these three methods had different problems in calculating the initial relaxation modulus. In this section, the applicability of the three methods to calculate the initial relaxation modulus is discussed.

In the midpoint method, the relaxation modulus in a time interval is assumed to be a fixed value, which is used to obtain the approximate value. To solve the relaxation modulus at the initial time, the length of the time interval, [*t_i_*, *t_i_*_+1_], must be narrow, which would increase the number of intervals and the calculation time. The Gauss integral is implemented to solve Equation (14) and the initial relaxation modulus and calculation time at different time intervals are represented in Table 9. It is obvious that with the decrease in the length of the time interval, the relaxation modulus of the inverse calculation increased gradually, which is closer to the initial relaxation modulus of *t* = 0. It can be seen from the results that its calculation time is more than 2000 s when the time interval is 0.0001 s. Therefore, the midpoint method should take considerable time to calculate the approximate value of the initial relaxation modulus.

The approximate interconversion method may be a good method to implement the interconversion of the relaxation modulus and creep compliance. However, it obtained an approximate result by simplifying the power law model. It can be seen clearly from Equation (15) that the approximate interconversion method removed the elastic parameters of the power law model. If the elastic parameter is retained, the relaxation modulus in the Laplace domain is as shown in Equation (27). In this way, the time–domain solution of the relaxation modulus could not be obtained by the Laplace transform. The initial relaxation modulus calculated by Equation (20) is equal to zero when *t* = 0, which is completely inconsistent with reality. Thus, the approximate interconversion method is unable to calculate the initial relaxation modulus.

(27) $$E(s)=\frac{1}{as+b\frac{\Gamma (m+1)}{s^{m-1}}}$$

The Laplace transform method can quickly calculate the relaxation modulus at any time. Similarly, the Laplace transform method cannot calculate the relaxation modulus at the initial time. It is obvious from Equation (23) that parameters *r* and s(θ) are equal to infinity when *t* is equal to zero. Consequently, the Laplace transform method is also unable to obtain the initial relaxation modulus.

## 5. The Proposed Method

It is seen from the analysis in Section 4 that the approximate interconversion method and Laplace transform method cannot obtain the initial relaxation modulus, and the midpoint method can gain the approximate value with a longer calculation time. The difference between the approximate value and the exact value of the initial relaxation modulus is unknown. In this part, a method is proposed in which the exact solution for the initial relaxation modulus is obtained from creep compliance by the initial value theorem of the Laplace transform. The initial value theorem of the Laplace transform [26] is as follows:

(28) $$\underset{t\to 0}{\lim}f(t)=\underset{s\to \infty }{\lim}sF(s)$$

where *f*(*t*) is the function in the time domain and *F*(*s*) is the function of *f*(*t*) in the Laplace domain.

Equation (28) is the fundamental theorem of Laplace transform, which indicates that the value of the time domain at time *t* = 0 can be solved by approaching infinity in the Laplace domain *s*, and it is suitable for solving the relaxation modulus value at the initial time *t* = 0. When the Prony series is used as the creep compliance model, the relaxation modulus at the initial time is calculated according to the following formula:

(29) $$\begin{array}{ll} \underset{t\to 0}{\lim}f(t)=\underset{s\to \infty }{\lim}sF(s) & =\underset{s\to \infty }{\lim}sE(s)\\ & =\underset{s\to \infty }{\lim}\frac{s}{J_{0}s+\sum_{i=1}^{7}J_{i}(s-\frac{\tau_{i}s^{2}}{\tau_{i}s+1})}\\ & =\underset{s\to \infty }{\lim}\frac{1}{J_{0}+\sum_{i=1}^{7}J_{i}(1-\frac{\tau_{i}s}{\tau_{i}s+1})}\\ & =\frac{1}{J_{0}} \end{array}$$

When the power law is used as the creep compliance model, the relaxation modulus at the initial time is calculated according to the following formula:

(30) $$\begin{array}{ll} \underset{t\to 0}{\lim}f(t)=\underset{s\to \infty }{\lim}sF(s) & =\underset{s\to \infty }{\lim}sE(s)\\ & =\underset{s\to \infty }{\lim}\frac{s}{as+b\frac{\Gamma (m+1)}{s^{m-1}}}\\ & =\underset{s\to \infty }{\lim}\frac{1}{a+b\frac{\Gamma (m+1)}{s^{m}}}\\ & =\frac{1}{a} \end{array}$$

Since the parameter *J*_0_ of the Prony series is equal to the parameter *a* of the power law, Equation (29) or Equation (30) could be used to calculate the initial relaxation modulus, and the results are shown in Table 10. Compared with the initial relaxation modulus *E*_0.00005s_ of SMA-13, AC-20, and ATB-25 at 25 °C and 0.1 MPa calculated by the midpoint method, the error is less than 2%, which indicated that *E*_0.00005s_ can be used to calculate the initial relaxation modulus. However, the midpoint method should take considerable time.

## 6. Extended to Other Viscoelastic Models

The purpose of this section is to extend the above research results to other viscoelastic constitutive models. The accurate characterization of the mechanical properties is of great significance to the evaluation and selection of asphalt and asphalt mixtures. A number of viscoelastic models have been used to model the mechanical properties of rheological materials, such as the classical viscoelastic model [6], 2S2P1D [38], and Huet [39]. The Prony series is the simplified generalized Kelvin model, and the creep compliance mathematical forms of the 2S2P1D and Huet models are shown in Equations (31) and (32), respectively.

(31) $$D(t)=\frac{1}{E_{\infty }}[1+\delta \frac{{\left(t/\tau \right)}^{k}}{\Gamma (k+1)}+\frac{{\left(t/\tau \right)}^{h}}{\Gamma (h+1)}+\frac{t}{\eta }]$$

(32) $$D(t)=\frac{1}{E_{\infty }}[1+\delta \frac{{\left(t/\tau \right)}^{k}}{\Gamma (k+1)}+\frac{{\left(t/\tau \right)}^{h}}{\Gamma (h+1)}]$$

where D(t) is the creep compliance, $E_{\infty }$ is the glassy modulus, *h* and *k* are exponents, δ is the dimensionless constant, *t* is load time.

The 2S2P1D model was selected to demonstrate the whole derivation process of the initial relaxation modulus. First, the Laplace transform is applied to Equation (31) and the creep compliance in the Laplace domain is obtained as follows:

(33) $$D(s)=\frac{1}{sE_{\infty }}+\frac{\delta E_{\infty }}{\tau^{k}s^{k+1}}+\frac{E_{\infty }}{\tau^{h}s^{h+1}}+\frac{E_{\infty }}{\eta s^{2}}$$

Then, the relaxation modulus in the Laplace domain can be derived by substituting Equation (33) into Equation (11):

(34) $$E(s)=\frac{1}{\frac{s}{E_{\infty }}+\frac{\delta E_{\infty }}{\tau^{k}s^{k-1}}+\frac{E_{\infty }}{\tau^{h}s^{h-1}}+\frac{E_{\infty }}{\eta }}$$

Finally, the initial relaxation modulus in the time domain can be obtained by utilizing the Laplace initial value theorem and Equation (34) is obtained:

(35) $$\begin{array}{ll} \underset{t\to 0}{\lim}E(t)=\underset{s\to \infty }{\lim}sE(s) & =\underset{s\to \infty }{\lim}\frac{s}{\frac{s}{E_{\infty }}+\frac{\delta E_{\infty }}{\tau^{k}s^{k-1}}+\frac{E_{\infty }}{\tau^{h}s^{h-1}}+\frac{E_{\infty }}{\eta }}\\ & =\underset{s\to \infty }{\lim}\frac{1}{\frac{1}{E_{\infty }}+\frac{\delta E_{\infty }}{\tau^{k}s^{k}}+\frac{E_{\infty }}{\tau^{h}s^{h}}+\frac{E_{\infty }}{\eta s}}\\ & =E_{\infty } \end{array}$$

when rheological models were used to study the conversion between creep compliance and relaxation modulus, the initial relaxation modulus could be calculated according to Table 11.

When a new viscoelastic model is used to convert the relaxation modulus from creep compliance, the initial relaxation modulus can be obtained as follows: (1) derive the creep model in the Laplace domain by applying the Laplace transform to the creep model in the time domain. (2) Derive the relaxation modulus in the Laplace domain by using the interconversion between the creep compliance and relaxation modulus in the Laplace domain. (3) Derive the initial relaxation modulus by utilizing the Laplace initial value theorem. This method is suitable for any rheological material to obtain the initial relaxation modulus.

## 7. Conclusions

In this paper, the linear viscoelastic theory is reviewed, and creep experiments of asphalt mixtures are conducted to establish the creep model. Then, the practicability of several conversion methods for solving the initial relaxation modulus is discussed, and a new elementary formula for the initial relaxation modulus from the creep compliance is proposed by Laplace initial value theorem. Finally, the calculation methods of the initial modulus for several common rheological models are proposed. The following conclusions can be drawn from this study:

(1) The midpoint method, approximate interconversion method, and Laplace transform method are three kinds of methods for conversion between creep compliance and relaxation modulus. The approximate interconversion method and Laplace transform method can be unable to calculate the initial relaxation modulus. The midpoint method can calculate the approximate value of the initial relaxation modulus with a very small interval length (approximately 0.0001 s) and requires a long computation time.
(2) The relaxation modulus in the Laplace domain can be derived by the interconversion between the creep compliance and relaxation modulus. A new elementary formula for the initial relaxation modulus, which is obtained by the Laplace initial value theorem, is proposed to calculate the initial relaxation modulus. The calculation result of the proposed method can be verified by the midpoint method with an error of less than 2%.
(3) The proposed method was extended to other common rheological models (power law, 2S2P1D and Huet models), and the expressions of the initial relaxation modulus were derived. The proposed method is suitable for any rheological model to determine the initial relaxation modulus from creep compliance.

This paper adopts viscoelastic theory to calculate the initial relaxation modulus, and there is no validation of relaxation test results. In the future, the relaxation test results will be used for verification (currently, there is no good testing instrument). This research approach will be applied to the conversion of dynamic viscoelastic parameters.

## Figures and Tables

**Table 1:** Asphalt properties.

Asphalt	Penetration (25 °C), 0.1 mm	Ductility (5 °C), (cm)	Softening Point (°C)
No. 90	87.5	>100	45.9
SBS-modified asphalt	47.3	50	79.3
SBS rubber compound modified asphalt	37.1	11.6	75.6

**Table 2:** Gradation of three asphalt mixtures.

Type		Sieve Size (mm)						
		31.5	26.5	19	16	13.2	9.5	4.75
SMA-13	Passing (%)	—	—	—	100	95	62.5	29
AC-20		—	100	95	85	71	61	41
ATB-25		100	95	70	58	52	42	30
		2.36	1.18	0.6	0.3	0.15	0.075	—
SMA-13	Passing (%)	23	22.5	16	11	8.5	5	—
AC-20		30	26.5	18.5	12.5	9.5	6.5	—
ATB-25		23.5	17.5	13	9.5	6.5	4	—

**Table 3:** Three types of asphalt mixtures.

Mixture Type	Gradation Type	Asphalt Binder		Air Void (%)
		Type	Optimum Asphalt Content (%)	
type-A	SMA-13	SBS rubber compound modified asphalt	6.0	3.5 ± 0.5
type-B	AC-20	SBS-modified asphalt	4.5	4.5 ± 0.5
type-C	ATB-25	No. 90 asphalt	3.8	5.0 ± 0.5

**Table 4:** Values of retardation times $\tau_{i}$.

*i*	1	2	3	4	5	6	7
$\tau_{i}$	0.001	0.01	0.1	1	10	100	1000

**Table 5:** Prony series coefficients of the three types of asphalt mixtures at 20 °C.

Coefficient	SMA-13		AC-20		ATB-25	
	0.1 MPa	0.3 MPa	0.1 MPa	0.3 MPa	0.1 MPa	0.3 MPa
*J*_0_	0.0149	0.01057	0.02045	0.03072	0.02559	0.03028
*J*_1_	0.003881	0.003181	0.002441	0.0008284	0.001178	0.003868
*J*_2_	0	0.003137	0.002394	0.0001381	0.015	0.005569
*J*_3_	0.01313	0.001591	0.001264	0.0002906	0.01305	0.006104
*J*_4_	0.003267	0.002623	0.002111	0.002463	0.02617	0.006488
*J*_5_	0.0111	0.01262	0.07234	0.003178	0.02103	0.002301
*J*_6_	0.003504	0.0007689	0.003217	0.009393	0.0001095	0.004382
*J*_7_	0.000726	0.01096	0.01535	0.007306	0.06846	0.004295
*R* ^2^	0.985	0.999	0.999	0.998	0.951	0.996

**Table 6:** Prony series coefficients of the three types of asphalt mixtures at 35 °C.

Coefficient	SMA-13		AC-20		ATB-25	
	0.1 MPa	0.3 MPa	0.1 MPa	0.3 MPa	0.1 MPa	0.3 MPa
*J*_0_	0.02133	0.01359	0.08657	0.03278	0.04601	0.04051
*J*_1_	0.00103	0.001	0.001038	0.001785	0.02481	0.02478
*J*_2_	0	0.0003397	0.001118	0.0006723	0.01796	0.03427
*J*_3_	0.008726	0.0006738	0.001928	0.0003776	0.01696	0.009355
*J*_4_	0.01949	0.0009308	0.006294	0.0004823	0.02721	0.005907
*J*_5_	0.02676	0.01654	0.002792	0.001346	0.00836	0.008974
*J*_6_	0.001955	0.0009805	0.006368	0.004083	0.003623	0.004281
*J*_7_	0.001481	0.0109	0.007322	0.002924	0.008508	0.007547
*R* ^2^	0.999	0.999	0.999	0.993	0.993	0.991

**Table 7:** Power law coefficients of the three types of asphalt mixtures at 20 °C.

Coefficient	SMA-13		AC-20		ATB-25	
	0.1 MPa	0.3 MPa	0.1 MPa	0.3 MPa	0.1 MPa	0.3 MPa
*a*	0.0149	0.01057	0.02045	0.03072	0.02559	0.03028
*b*	0.01484	0.01712	0.05557	0.003497	0.05809	0.02044
*m*	0.08164	0.07876	0.08628	0.2919	0.07942	0.06499
*R* ^2^	0.85	0.896	0.729	0.997	0.932	0.999

**Table 8:** Power law coefficients of the three types of asphalt mixtures at 35 °C.

Coefficient	SMA-13		AC-20		ATB-25	
	0.1 MPa	0.3 MPa	0.1 MPa	0.3 MPa	0.1 MPa	0.3 MPa
*a*	0.02133	0.01359	0.08657	0.03278	0.04601	0.04051
*b*	0.04624	0.01273	0.008806	0.002628	0.08857	0.07618
*m*	0.04464	0.1082	0.1545	0.2272	0.02291	0.02813
*R* ^2^	0.708	0.858	0.999	0.995	0.976	0.975

**Table 9:** Initial relaxation modulus and calculation time in different time intervals (20 °C, 0.1 MPa).

Mixture	Analysis Index	Time Interval (s)							
		2	1	0.8	0.4	0.1	0.01	0.001	0.0001
		*E*_1s_	*E*_0.5s_	*E*_0.4s_	*E*_0.2s_	*E*_0.05s_	*E*_0.005s_	*E*_0.0005s_	*E*_0.00005s_
SMA	Value (MPa)	29.2	30.9	31.5	33.9	42.0	52.5	61.0	66.2
	Calculating time (s)	0.117	0.223	0.297	0.512	2.14	11.9	204.1	2134.2
AC	Value (MPa)	29.0	32.6	33.5	35.7	38.5	42.3	46.6	48.6
	Calculating time (s)	0.098	0.208	0.265	0.834	2.13	14.3	201.5	2095.4
ATB	Value (MPa)	14.1	15.6	16.1	17.8	21.5	30.4	37.3	38.9
	Calculating time (s)	0.118	0.241	0.339	0.528	2.01	15.3	197.6	2183.4

**Table 10:** Initial relaxation modulus of asphalt mixtures at different temperatures and loads (MPa).

Asphalt Mixture	SMA-13		AC-20		ATB-25	
Load	0.1 MPa	0.3 MPa	0.1 MPa	0.3 MPa	0.1 MPa	0.3 MPa
20 °C	67.1	94.6	48.9	32.6	39.1	33.0
35 °C	46.9	73.6	11.6	30.5	21.7	24.7

**Table 11:** Initial relaxation modulus of different viscoelastic models (MPa).

Rheological Models	Initial Relaxation Modulus
Prony series	1/*J*_0_
Power law	1/*a*
2S2P1D	$E_{\infty }$
Huet	$E_{\infty }$

## Data Availability

All data generated or analyzed during this study are included in this published article.
